# Prediction of a Competing Endogenous RNA Co-expression Network by Comprehensive Methods in Systemic Sclerosis-Related Interstitial Lung Disease

**DOI:** 10.3389/fgene.2021.633059

**Published:** 2021-07-05

**Authors:** Yue-Mei Yan, Ji-Na Zheng, Li-Wei Wu, Qian-Wen Rao, Qiao-Rong Yang, Di Gao, Qiang Wang

**Affiliations:** ^1^Department of Dermatology, Zhongshan Hospital, Fudan University, Shanghai, China; ^2^Department of Thoracic Surgery, Shanghai Public Health Clinical Center, Fudan University, Shanghai, China; ^3^Minhang Branch, Zhongshan Hospital, Fudan University, Shanghai, China

**Keywords:** systemic sclerosis, weighted correlation network analysis, competing endogenous RNA network, SNHG16, Gene Expression Omnibus

## Abstract

Systemic sclerosis (SSc) is an immune-mediated connective tissue disease characterized by fibrosis of multi-organs, and SSc-related interstitial lung disease (SSc-ILD) is a leading cause of morbidity and mortality. To explore molecular biological mechanisms of SSc-ILD, we constructed a competing endogenous RNA (ceRNA) network for prediction. Expression profiling data were obtained from the Gene Expression Omnibus (GEO) database, and differential expressed mRNAs and miRNAs analysis was further conducted between normal lung tissue and SSc lung tissue. Also, the interactions of miRNA–lncRNA, miRNA–mRNA, and lncRNA–mRNA were predicted by online databases including starBase, LncBase, miRTarBase, and LncACTdb. The ceRNA network containing 11 lncRNAs, 7 miRNAs, and 20 mRNAs were constructed. Based on hub genes and miRNAs identified by weighted correlation network analysis (WGCNA) method, three core sub-networks—SNHG16, LIN01128, RP11-834C11.4(LINC02381)/hsa-let-7f-5p/IL6, LINC01128/has-miR-21-5p/PTX3, and LINC00665/hsa-miR-155-5p/PLS1—were obtained. Combined with previous studies and enrichment analyses, the lncRNA-mediated network affected LPS-induced inflammatory and immune processes, fibrosis development, and tumor microenvironment variations. The ceRNA network, especially three core sub-networks, may be served as early biomarkers and potential targets for SSc, which also provides further insights into the occurrence, progression, and accurate treatment of SSc at the molecular level.

## Introduction

Systemic sclerosis (SSc), also named scleroderma, is an immune-mediated connective tissue disease characterized by fibrosis of the skin and internal organs with unknown etiology (The Lancet, 2017). It is believed to be related to the interplay between vascular damage, immunological pathways, and connective tissue repair. The development of progressive systemic fibroproliferative process characteristic is crucial in the pathogenesis of SSc. According to the European Scleroderma Trials and Research (EUSTAR) database, pulmonary fibrosis accounted for 35% of disease-specific mortality and about 20% of overall mortality, which indicates interstitial lung disease (ILD), a leading cause of morbidity and mortality (Tyndall et al., 2010).

Recent theoretical and experimental studies have suggested that non-coding RNAs (ncRNAs), such as long non-coding RNAs (lncRNAs) and microRNAs (miRNAs), played important roles in the progression of SSc (Wang et al., 2016; Angiolilli et al., 2018; Henry et al., 2019; Wasson et al., 2020). In general, the combination of miRNA and mRNA will lead to the downregulation of mRNA gene expression. The competing endogenous RNA (ceRNA) hypothesis posits that specific ncRNAs, such as lncRNAs and circular RNAs (circRNAs), can impair miRNA activity through occupying the binding sites on them, thereby upregulating mRNA gene expression (Thomson and Dinger, 2016). In another way, lncRNAs act as molecular sponges to attract miRNAs, contributing to various human disease processes. The ceRNA hypothesis has been proved to be implicated in the development of various cancers and diseases by more and more researches, including other autoimmune diseases (Liu et al., 2020; Wang J. et al., 2020). However, the ceRNA hypothesis about SSc has not been proposed so far.

To further explore the biological functions of ncRNAs at the molecular level in SSc, we constructed a ceRNA co-expression network for some clues. In this study, we obtained expression profiling data from the NCBI Gene Expression Omnibus (GEO)^1^ dataset and compared the expression profiles between 15 SSc-ILD patients and 5 healthy controls (HCs). Differential expressed miRNAs were used to predict targeted mRNAs and lncRNAs through several online databases, including starBase, LncBase, miRTarBase, and LncACTdb. Weighted correlation network analysis (WGCNA) (Langfelder and Horvath, 2008), an R package for WGCNA, has been previously successfully applied in various biological contexts to reveal the relationship between modules and clinical features. WGCNA was applied to detect key miRNAs and hub genes, which played significant roles in the pathological process of SSc. Finally, we identified 11 lncRNAs, 7 miRNAs, and 20 mRNAs to construct a lncRNA–miRNA–mRNA ceRNA network. Among the network, three core sub-networks, SNHG16, LIN01128, RP11-834C11.4(LINC02381)/hsa-let-7f-5p/IL6, LINC01128/has-miR-21-5p/PTX3, and LINC00665/hsa-miR-155-5p/PLS1, were considered to be of huge significance for the pathological development in SSc, by influencing LPS-induced inflammatory and immune processes, fibrosis development, and tumor microenvironment variations (Shah and Rosen, 2011; De Luca et al., 2015; Sambataro et al., 2015; Maria et al., 2018).

To the best of our knowledge, this is the first study to focus on the prediction of ceRNA network in SSc and SSc-ILD, providing new perspectives on SSc pathogenesis, progression, and treatment.

## Materials and Methods

### Data Collection

**FIGURE 1:**
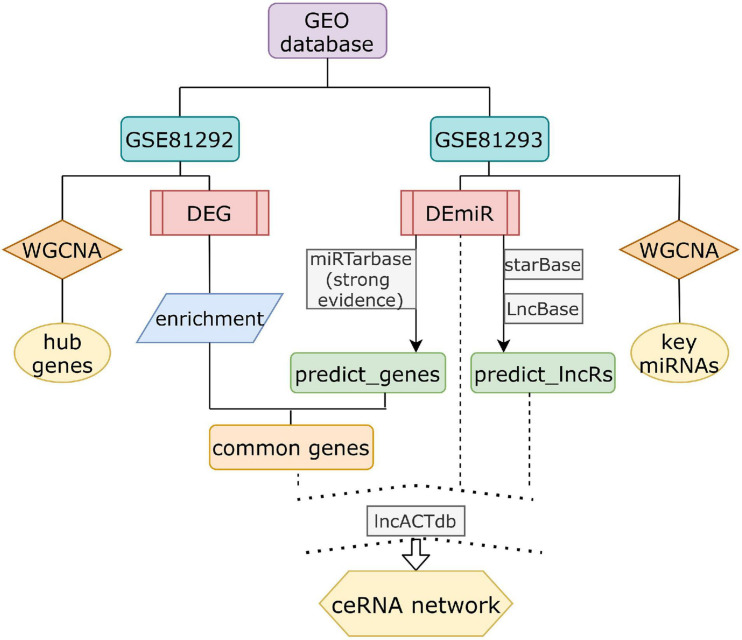
The schematic diagram of the data analysis. DEG, differential expressed genes; DEmiRs, differential expressed miRNAs.

Total expression profiling datasets of SSc-ILD were obtained from GEO, a public data repository. Screening was performed in accordance with the following criteria: (1) at least 10 SSc and health control (HC) samples were included for each gene expression dataset; replication or drug-trail or cell lines samples should be excluded; (2) tissues originate from lung biopsy on *Homo sapiens*; and (3) datasets containing both non-coding RNA expression and mRNA expression were included. Finally, only dataset GSE81294 (Christmann et al., 2016) met all these criteria mentioned above. Dataset GSE81294 was composed of two sub-datasets [GSE81292 (Christmann et al., 2016) and GSE81293 (Christmann et al., 2016)] from 15 SSc-ILD samples (from 11 patients) and 5 matched HC samples (from four controls). Patients included were classified as having diffuse SSc (dSSc) (*n* = 7) and limited SSc (lcSSc) (*n* = 4). All patients with SSc-related ILD were female, and HC samples came from three female controls and one male control. The specimens were obtained prior to treatment with disease-modifying drugs. The biopsy time from diagnosis again was unclear. GSE81292, based on the platform of GPL18991 Affymetrix Human Genome U133A 2.0 Array, investigated mRNA expression profile. GSE81293, based on the platform of GPL16384 Affymetrix Multispecies miRNA-3 Array, investigated miRNA expression profile. The flowchart of our design is shown in Figure 1.

### Differential Expression Analysis

Differently expressed mRNAs (DEGs) and microRNAs (DEmiRNAs) were retrieved using the R package “limma” in the environment of R software (version 4.0.2). *P*-value < 0.05 and | log2FC (Fold change)| > 1 were set as the thresholds for identifying DEGs and DEmiRNAs. To visualize the results, volcano plots and heatmap plots were generated using the R package “limma” and “pheatmap.”

### Functional Enrichment Analysis of DEGs

Gene Ontology (GO) analysis (Wang et al., 2019) and Kyoto Encyclopedia of Genes and Genomes (KEGG) analysis were performed to identify functional and molecular features of DEGs. The biological processes (BP), cell components (CC), molecular function (MF), and KEGG pathways were retrieved with a cutoff criterion of *P* < 0.05 and visualized by the R packages “enrichplot” and “GOplot.”

### Construction of a ceRNA Network

DEmiRNAs in dataset GSE81293 were selected to be further studied. First, miRNA–lncRNA interactions were predicted utilizing starBase (v2.0) (Li et al., 2014), LncBase Predicted v.2 (threshold = 0.9), and LncBase Experimental v.2 computational methods (Paraskevopoulou et al., 2013). Next, miRNA–mRNA interactions were obtained from miRTarBase (Chou et al., 2018), a manually curated database that provides experimentally supported miRNA–gene interactions. Only high-confidence functional miRNA–gene interactions supported by reporter assay and/or Western blot data were retained and used to intersect with DEGs of GSE81292. Then, predicted miRNA–lncRNA interactions and miRNA–mRNA interactions were further filtered by lncACTdb (Wang et al., 2019), an updated database of experimentally supported ceRNA interactions curated from low- and high-throughput experiments. Finally, the intersections of predicted lncRNAs and mRNAs, respectively, were visualized in venn plots, and a ceRNA network was visualized in a Sankey plot by the R package “ggalluvial.”

### Weighted Correlation Network Analysis

The R package “WGCNA” was adopted to construct gene co-expression networks and detect the disease-related modules and key miRNAs and hub genes on the R platform. Top 25% most variant genes in GSE81292 and GSE81293 were selected for analysis. By setting soft-thresholding power, a scale-free network was constructed. Topological overlap measurement (TOM) represented the overlap in the shared neighbors for further identifying key modules. The weighted co-expression relationship in the adjacency matrix was assessed by the Pearson correlation and adjusted. *P* < 0.05 was considered significant. GO enrichment analysis was applied in order to explain the biological role of the key modules with the R package “enrichplot.”

## Results

### Differential Gene Expression From GEO Was Analyzed

**FIGURE 2:**
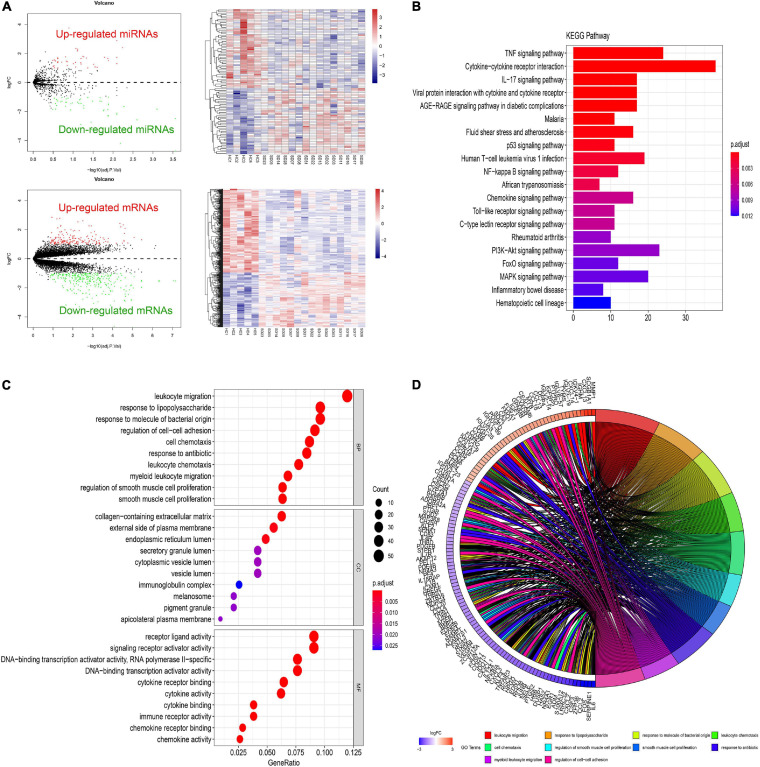
Differential expressed gene profiles analysis. **(A)** Volcano and heatmap plots of DEGs and DEmiRs in GSE81293 and GSE81292. **(B)** The top 10 KEGG enrichment pathways of DEGs in GSE81292. The length of each bar means the amounts of genes that belong to one certain pathway. The different color of each bar means *P*-adjust-value. **(C)** Top 10 biological process (BP), cellular component (CC), and molecular functions (MF) terms of DEGs in GSE81292. The size of each circle means the amounts of genes. The different color of each circle means *P*-adjust-value. GeneRatio means the ratio of genes that belong to this pathway divided by the number of genes in the background gene cluster that belong to this pathway. **(D)** GOchord plot of DEGs in GSE81292. DEG, differential expressed genes; DEmiRs, differential expressed miRNAs; KEGG, Kyoto Encyclopedia of Genes and Genomes; GO, gene ontology.

Different gene expression analysis of GSE81292 and GSE81293 was visualized with volcano plots and heatmap plots (Figure 2A). In total, 92 DElncRNAs (75 significantly downregulated and 17 significantly upregulated) and 465 DEGs (281 significantly downregulated and 184 significantly upregulated) were identified with | log2FC| > 1 and *P*-value < 0.05.

### Functional Enrichment Analysis of DEGs

As shown in Figures 2C,D, enriched GO-BP terms were mainly about inflammatory and immune processes, including “leukocyte migration,” “response to lipopolysaccharide,” “response to molecule of bacterial origin,” “regulation of cell–cell adhesion,” and “cell chemotaxis.” For GO-CC, enriched terms were generally involved in “collagen-containing extracellular matrix,” “external side of plasma membrane,” and “endoplasmic reticulum lumen.” Enriched GO-MF terms were mainly about “receptor ligand activity,” “signaling receptor activator activity,” and “DNA-binding transcription activator activity, RNA polymerase II-specific.” The results of KEGG enrichment were roughly about “TNF signaling pathway,” “cytokine–cytokine receptor interaction,” and “IL-17 signaling pathway” (Figure 2B). These most significantly enriched GO terms and KEGG pathways indicated the interactions of differentially expressed mRNAs at the functional level.

### Construction of a ceRNA Network

**FIGURE 3:**
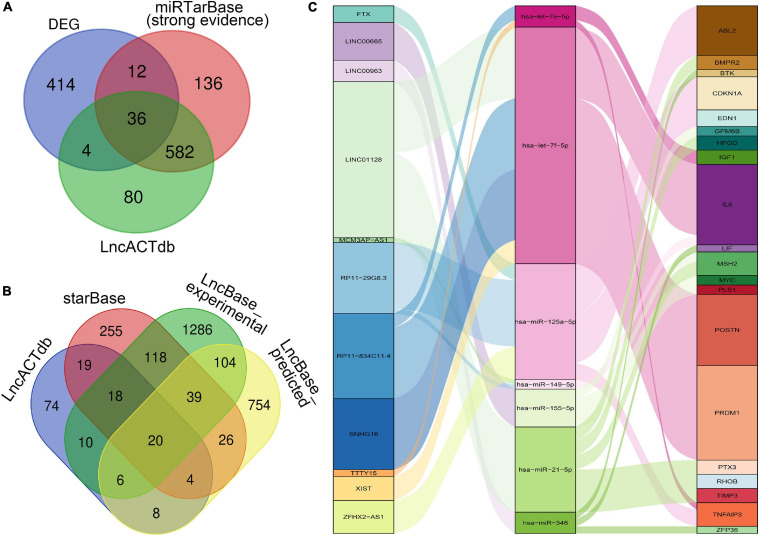
Construction of the ceRNA Co-expression network. **(A)** Venn plot: The intersection of DEGs, predicted mRNAs by miRTarBase, and predicted mRNAs by LncACTdb. **(B)** Venn plot: Overlapped lncRNAs predicted by starBase, LncBase_predicted, LncBase_experimental, and LncACTdb. **(C)** The lncRNA–miRNA–mRNA ceRNA Sankey plot was constructed *via* 11 lncRNAs, 7 miRNAs, and 20 mRNAs for SSc-ILD.

As shown in the Figures 3A,B, through the intersections obtained by the above database prediction, we screened 20 lncRNAs and 36 mRNAs in total. mRNAs or lncRNAs without predicted miRNA intersections were discarded. Besides, considering that there is a certain relationship between lncRNAs and mRNAs, those that are not related will also be filtered by the lnACTdb dataset. Finally, a ceRNA co-expression network consisting of 11 lncRNAs, 7 miRNAs, and 20 mRNAs was built after merging these predicted results and visualized by Sankey plot (Figure 3C).

In the network, hsa-let-7f-5p has four related lncRNAs, including RP11-834C11.4, SNHG16, LINC01128, and XIST. hsa-miR-21-5p has six targeted genes, including *TIMP3*, *PTX3*, *HPGD*, *BMPR2*, *RHOB*, and *MSH2*. LncRNA LINC01128 and mRNA *IL6* gained the most ceRNA relationship pairs, respectively.

### WGCNA Analysis

**FIGURE 4:**
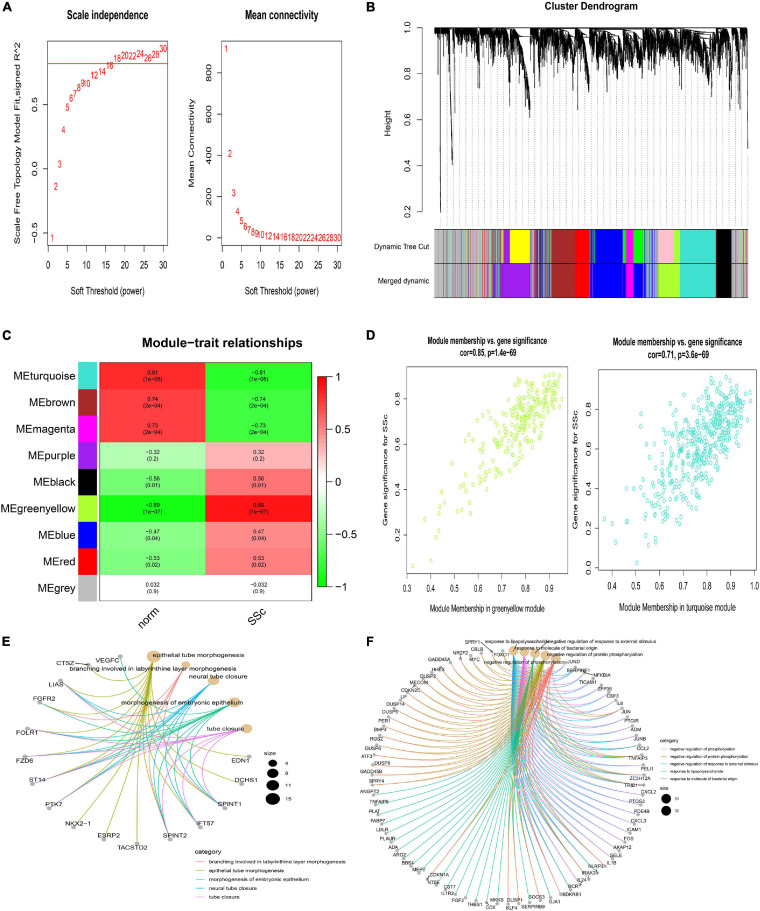
Construction of weighted gene co-expression network of GSE81292. **(A)** Analysis of the scale-free topology model fit index for soft threshold powers (β) and the mean connectivity for soft threshold powers. **(B)** Cluster dendrogram of the co-expression network modules was produced based on topological overlap in the mRNAs. Each branch means one gene. Each color means one co-expressed module. Twelve modules were merged into nine modules. **(C)** Heatmap of the correlation between module eigengenes and clinical traits. *P*-value is shown in each color cell coded by the correlation between modules and traits (red indicates positive correlation, while green indicated negative correlation). **(D)** Scatter plot of module eigengenes in the greenyellow and turquoise module. **(E)** GO enrichment of genes in the greenyellow module. Each color of lines means one enrichment category for genes. **(F)** GO enrichment of genes in the turquoise module. SSc, systemic sclerosis; GO, gene ontology.

Weighted correlation network analysis was applied to seek for key modules and hub genes in GSE81292. Gene modules were analyzed among the first 25% mRNAs by variance comparison. The soft-thresholding power was selected as 18 to identify co-expressed gene modules (Figure 4A). Based on topological overlap matrix (TOM) dissimilarity algorithm, 12 co-expression modules were finally constructed and then merged into 9 modules (Figure 4B). Module–trait correlations analysis showed that multiple modules were related to SSc (Figure 4C). Clearly, the greenyellow module (*r* = 0.89, *p* = 1e–07) was of the most importance among them, followed by the turquoise module (*r* = –0.81, *p* = 1e–05). The correlation values between module membership (MM) and gene significance (GS) for SSc were 0.85 and 0.71 in the greenyellow module and turquoise module, respectively (Figure 4D), which indicated strong relativity. As shown in Figure 4E, genes in the greenyellow module were mainly enriched in epithelial or tube, such as “epithelial tube morphogenesis,” “neural tube closure,” “morphogenesis of embryonic epithelium,” and so on. Genes in the turquoise module were roughly enriched in inflammation or stimulus, such as “negative regulation of phosphorylation,” “negative regulation of response to external stimulus,” “response to lipopolysaccharide,” and “response to molecule of bacterial origin” (Figure 4F).

**FIGURE 5:**
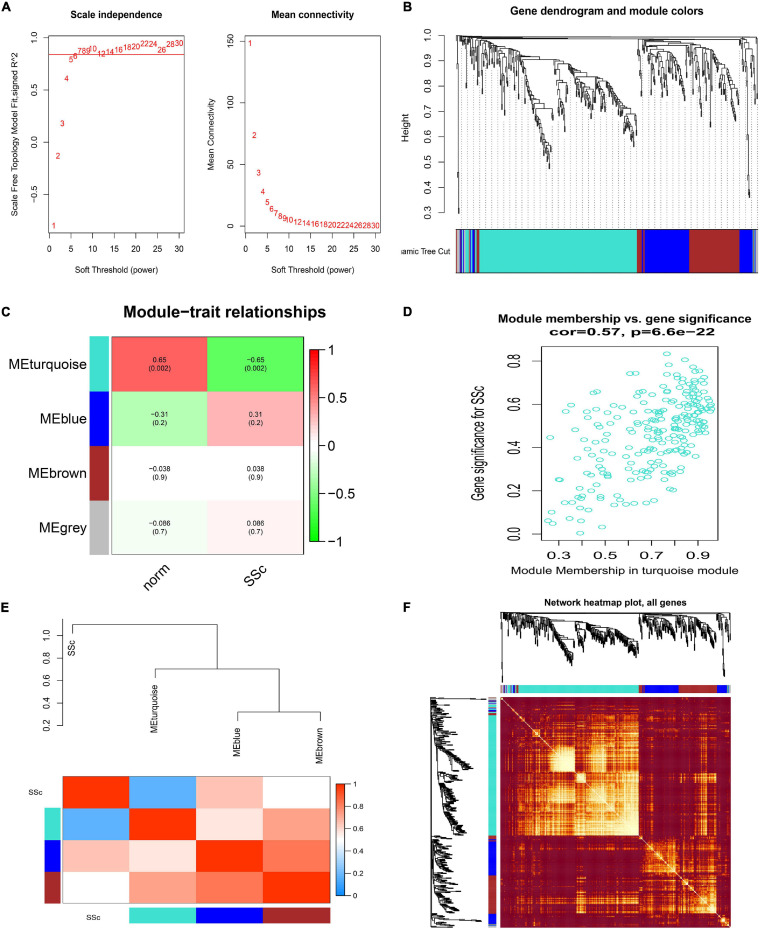
Construction of weighted gene co-expression network of GSE81293. **(A)** Analysis of the scale-free topology model fit index for soft threshold powers (β) and the mean connectivity for soft threshold powers. **(B)** Cluster dendrogram of the co-expression network modules was produced based on topological overlap in the miRNAs. Each branch means one miRNA. Each color means one co-expressed module. **(C)** Heatmap of the correlation between module eigengenes and clinical traits. **(D)** Scatter plot of module eigengenes in the turquoise module. **(E)** Dendrogram and unsupervised hierarchical clustering heatmap of module eigengenes and SSc. **(F)** Interactive relationship analysis of co-expression miRNAs. The light color indicates topological overlap, while the darker color indicates a high topological overlap. SSc, systemic sclerosis.

Similarly, when in GSE81293, the soft-thresholding power was chosen as 7 to identify four co-expressed gene modules (Figures 5A,B). Module–trait correlation analysis revealed that only the turquoise module (*r* = -0.65, *p* = 0.002) was considered to be statistically meaningful, and was most related to SSc (Figure 5C). The correlation value between module membership (MM) and gene significance (GS) for SSc was 0.57 in the turquoise module (Figure 5D), suggesting reasonable relativity. The dendrogram and adjacency heatmap of eigengenes (Figure 5E) further indicated that the turquoise module was closest to SSc traits. Figure 5F showed the interactive relationship analysis of co-expression miRNAs.

### Identification of Hub Genes and miRNAs

According to the value of intra-module connectivity, genes ranked in the top 30 were regarded as hub genes in the greenyellow and turquoise modules of GSE81292, which were summarized in Table 1.

**TABLE 1:** Top 30 Hub Genes in the light green and turquoise module of GSE81292.

Rank	Module greenyellow			Module turquoise		
	Gene name	GS	MM	Gene name	GS	MM
1	ITGA5	–0.794	–0.910	STC1	–0.842	0.984
2	ARMC1	0.834	0.918	SERPINE1	–0.885	0.981
3	GALNT12	0.767	0.945	GADD45B	–0.879	0.963
4	FPGT	0.825	0.917	FOSL2	–0.747	0.970
5	IDH1	0.869	0.927	BHLHE40	-0.835	0.955
6	ERP29	0.902	0.943	IL6#	-0.838	0.942
7	SLC35A5	0.836	0.892	BATF3	-0.759	0.945
8	NUP37	0.772	0.892	CHSY1	-0.794	0.943
9	TM9SF2	0.874	0.921	ZFP30	0.816	-0.949
10	HNMT	0.815	0.911	CSF3	–0.842	0.936
11	PDIA3	0.853	0.934	MT4	–0.842	0.940
12	CXADR	0.799	0.953	CCL2	–0.818	0.940
13	SMG8	0.762	0.919	MAFF	–0.865	0.924
14	PRKAG1	0.848	0.905	FKBP1A	–0.715	0.942
15	GANAB	0.873	0.901	RGS16	–0.860	0.925
16	PDGFB	–0.861	–0.922	NR4A3	–0.830	0.930
17	ST3GAL5	0.860	0.916	ZNF167	0.917	–0.924
18	RAB38	0.772	0.913	IL4R	–0.890	0.925
19	PIGF	0.784	0.911	MTMR4	0.849	–0.933
20	PLS1#	0.627	0.863	ITPKC	–0.832	0.928
21	PELO	–0.906	–0.901	SPSB1	–0.763	0.938
22	PTPN13	0.799	0.922	EHHADH	0.802	–0.931
23	GALNT7	0.823	0.924	MT2A	–0.852	0.926
24	CASD1	0.827	0.925	PTX3#	–0.617	0.934
25	CD9	0.648	0.866	SUV420H1	0.722	–0.926
26	DSG2	0.740	0.895	PTGIR	–0.829	0.921
27	TSEN2	0.838	0.905	FOSL1	–0.683	0.928
28	MSX1	–0.907	–0.919	C6orf130	0.741	–0.929
29	DBR1	0.847	0.887	XPO1	0.889	–0.913
30	THAP1	0.786	0.839	C5orf54	0.733	–0.927

*GS, gene significance; MM, module membership. “#” means that the gene is one of mRNAs in the ceRNA we constructed.*

**FIGURE 6:**
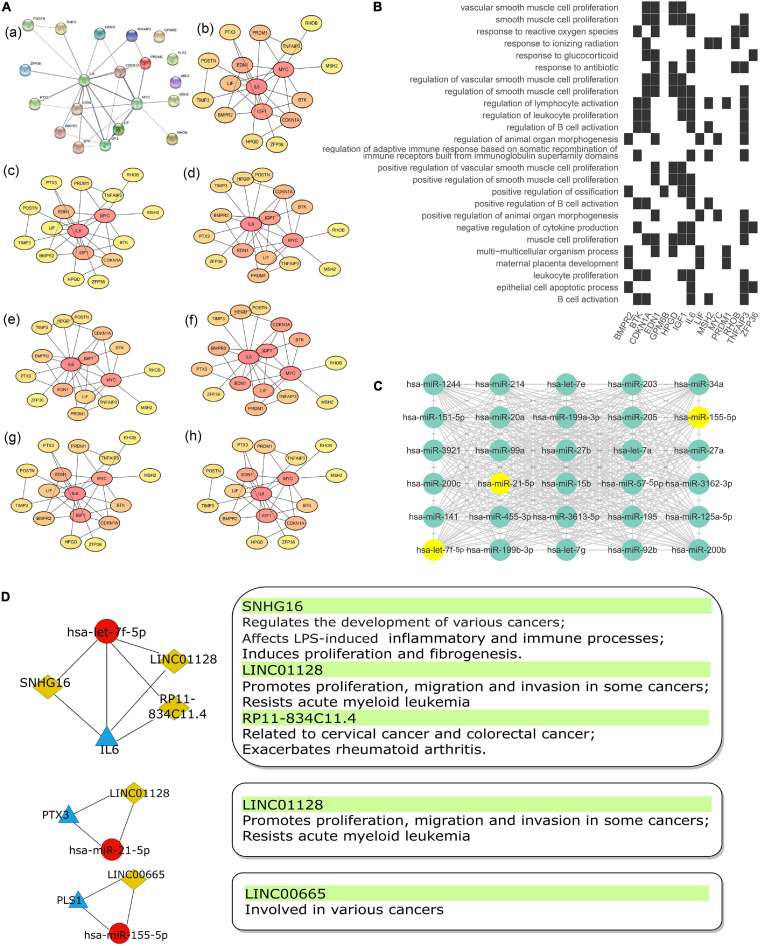
The Sub-network of lncRNA acting as a ceRNA to regulate miRNA–gene pairs in SSc. **(A)** PPI network **(a)** of 20 mRNAs in the ceRNA network were analyzed by “cytohubba” plug-in in cytoscape software with seven algorithm methods, including “Radiality” **(b)**, “Betweenness” **(c)**, “Closeness” **(d)**, “EPC” **(e)**, “MCC” **(f)**, “MNC” **(g)**, and “Degree” **(h)**. **(B)** Heatmap of enrichment analysis results of 20 mRNAs in ceRNA network. **(C)** Top 30 key miRNAs in the turquoise module of the co-expression network in GSE81293. Points where 30 key miRNAs overlap with miRNAs in ceRNA network are marked in yellow. **(D)** The sub-networks of let-7f-5p/IL-6 pair, miR-21-5p/PTX3 pair, and miR-155-5p/PLS1 pair. The left pipeline are the sub-networks of three experimentally supported miRNA–gene pairs in SSc and their linked lncRNAs. The right pipeline are the summarized functions of lncRNAs. PPI, protein–protein interaction; SSc, systemic sclerosis.

The top 30 most connected miRNAs in the turquoise module of GSE81293 were selected based on the calculated topological overlap value. These miRNAs were regarded as central roles in the pathogenesis of SSc. The exact results were input to cytoscape software (version 3.8.0) and visualized in Figure 6C.

### Sub-Networks

The intersection of 20 mRNAs in the ceRNA network and hub genes identified by WGCNA included *IL6*, *PLS1*, and *PTX3*. Also, their binding miRNAs (hsa-let-7f-5p, hsa-miR-21-5p, and hsa-miR-155-5p) were among the key miRNAs identified by WGCNA. Thus, we filtered three core sub-networks: SNHG16/LIN01128/RP11-834C11.4 (LINC02381)/hsa-let-7f-5p/IL6, LINC01128/hsa-miR-21-5p/PTX3, and LINC00665/hsa-miR-155-5p/PLS1 (Figure 6D). As shown in the protein–protein interaction (PPI) network (Figure 6A,a), *IL6* was in the center position. By utilizing the “cytohubba” plug-in in cytoscape software, the PPI network was further calculated by various methods, including “MNC,” “MCC,” “Radiality,” “EPC,” “Degree,” “Closeness,” and “Betweenness” (Figures 6A,b–h). *IL6* all ranked top 1, which indicated its significant role in the ceRNA sub-networks. *IL6* was mainly enriched in inflammatory and immune aspects, including “regulation of B cell activation,” “leucocyte proliferation,” “lymphocyte activation,” and so on (Figure 6B).

## Discussion

The triggering fibrosis processes involved in SSc are not clearly defined. Clinically, a variety of methods are used in disease treatment, such as corticosteroid, immunosuppressant therapy (Bournia et al., 2009), penicillamine (Jimenez and Sigal, 1991), traditional Chinese medicine, hematopoietic stem cell transplantation, and biological agents [Rituximab (Jordan et al., 2015) and Tocilizumab (Khanna et al., 2016)]. However, there is no so-called “best therapy” for all SSc patients. Recently, extensive evidence indicates that ncRNAs play critical roles in the regulation of the immune system and in autoimmune disease. The hsa-miR-27a-3p mediates reduction of the Wnt antagonist sFRP-1, and thus mediating fibrosis regression in SSc (Henderson et al., 2020). Moreover, long non-coding RNA H19X was found to be an obligatory factor for TGF-β-induced ECM synthesis as well as differentiation and survival of ECM-producing myofibroblasts (Pachera et al., 2020).

The newly discovered lncRNA-mediated regulation theory and network of ceRNA has improved our understanding of the precise molecular mechanism of many diseases, especially for cancers. For example, a lncRNA-associated ceRNA network was uncovered and systematically characterized global properties with prognostic value across 12 types of human cancer (Wang et al., 2015). However, ceRNA networks concerning autoimmune diseases or connective tissue diseases remain very rare. Consequently, there is an urgency exploring the regulatory mechanisms of ceRNAs and discovering novel biomarkers and therapeutic targets for SSc. In the present study, the ceRNA was composed of 7 miRNA nodes, 20 mRNA nodes, and 11 lncRNA nodes. Multiple databases were utilized to predict miRNA–lncRNA interactions and miRNA–mRNA interactions, including miRTarBase, starBase, and LncBase, which were based on experimentally supported evidence or computationally predicted methods. Due to the lack of lncRNA sequencing data of SSc-ILD, we also used LncACTdb to filter lncRNA–mRNA interactions and predict a ceRNA network. WGCNA co-expression analysis further identified core genes and miRNAs in the ceRNA. Through soft-threshold filtering, co-expression matrix constructing, weighted network establishing, and hierarchical clustering, module–trait networks can be constructed for understanding the clinical relevance of hub genes and key miRNAs accurately. Combined with comprehensive methods, we ultimately extracted three core sub-networks: SNHG16, LIN01128, RP11-834C11.4(LINC02381)/hsa-let-7f-5p/IL6, LINC01128/hsa-miR-21-5p/PTX3, and LINC00665/hsa-miR-155-5p/PLS1.

We summarized the functions of four lncRNAs in sub-networks as ceRNAs to regulate miRNA–gene interactions in SSc (Figure 6D). SNHG16, LIN01128, RP11-834C11.4(LINC02381) and LINC00665 were all verified to be involved in various cancers, such as osteosarcoma (Yao and Chen, 2020), cervical cancer (Chen et al., 2020), breast cancer (Ji et al., 2020), gastric cancer (Yang et al., 2020), and hepatic cancer (Ding et al., 2020). Besides, RP11-834C11.4(LINC02381) was proved to exacerbate rheumatoid arthritis (Wang and Zhao, 2020). Among them, SNHG16/hsa-let-7f/IL6 seems to be of importance particularly. A recent mechanistic investigation revealed that SHNG16 acts as a ceRNA to positively regulate Toll-like receptor 4 (*TLR4*) and thus affect the LPS-induced inflammatory and immune processes *via* mediating JNK and NF-κB pathways in pneumonia (Zhou et al., 2019). Besides, SNHG16 could induce proliferation and fibrogenesis *via* modulating miR-141-3p and *CCND1* in diabetic nephropathy (Jiang et al., 2020). Evidences also indicated that hsa-let-7f/IL6 were involved in immunity and fibrosis processes. MicroRNA let-7 is associated with the prognosis value of fibrotic diseases. Serum let-7 levels correlate with the severity of hepatic fibrosis (Matsuura et al., 2018) and expression levels of let-7 in skin correlate negatively with severity of pulmonary hypertension in SSc patients (Izumiya et al., 2015). As for *IL6* (interleukin 6), it is widely acknowledged that it encodes a cytokine that functions in inflammation and the maturation of B cells. In addition, the encoded protein is an endogenous pyrogen capable of inducing fever in people with autoimmune diseases or infections. *IL6* was also found to be involved in the fibrosis process and closely associated with SSc development. Spontaneous and stimulated IL-6 secretion by blood monocytes is elevated in SSc-ILD patients, compared with secretion by HC subjects (Crestani et al., 1994). Previous studies also indicate a potent profibrotic effect of *IL6 trans-*signaling *via* the JAK2/STAT3 and ERK pathways, supported by *in vitro* experiments (Khan et al., 2012). Besides, a study showed that let-7 directly regulated *IL6* expression by using the luciferase reporter system, and their relationship was verified by Western blot and real-time PCR (Dong et al., 2018). Taken together, we may infer that SNHG16 targets has-let-7f-5p/IL6 to participate in the occurrence of inflammation and the process of fibrosis in SSc-ILD. However, the underlying mechanism of the involvement of SNHG16 in SSc remains unclear. The LINC01128-mediated hsa-miR-21-5p/PTX3 pair was related to similar pathways as well. miR-21 was found to be significantly elevated in SSc fibroblasts and to regulate TGF-β-regulated fibrosis-related gene expression (Zhu et al., 2013) and collagen expression (Jafarinejad-Farsangi et al., 2019). *PTX3* (pentraxin 3) serves as a biomarker for several autoimmune diseases (Wu et al., 2020), participating in inflammation regulation and fibrocyte differentiation. *PTX3* was elevated in the serum (Shirai et al., 2015) and fibroblasts (Luchetti et al., 2004) of SSc patients. The overexpression of *PTX3* production from hyaluronan-stimulated fibroblasts is mediated by TLR4 signaling pathway due to enhanced oxidative stress in SSc (Iwata et al., 2009). Studies showed that the LINC00665/hsa-miR-155-5p/PLS1 pair might be involved in fibrosis. The expression of miR-155 was upregulated by inflammasome response in SSc-driven fibrosis (Artlett et al., 2017). miR-155 PBMC expression strongly correlated with lung function tests in SSc-ILD and miR-155ko mice developed milder lung fibrosis and survived longer in bleomycin-induced model (Christmann et al., 2016). *PLS1* (plastin 1) is one of the actin-binding protein family members that are conserved throughout eukaryote evolution. *PLS1* drives metastasis of colorectal cancer through the IQGAP1/Rac1/ERK pathway (Zhang et al., 2020) and promotes osteoblast differentiation by regulating intracellular Ca2+ (Wang L. et al., 2020).

In summary, this is the first study to try to construct a ceRNA network in SSc. Our study found out the key gene co-expression modules and hub genes and key miRNAs as well. Among them, three core sub-networks, SNHG16, LIN01128, RP11-834C11.4(LINC02381)/hsa-let-7f-5p/IL6, LINC01128/has-miR-21-5p/PTX3, and LINC00665/hsa-miR-155-5p/PLS1 may serve as promising prognostic predictor sand therapeutic targets for SSc-ILD. The advantage of our study is that we chose to analyze the data from the same tissue source to minimize the heterogeneity caused by different tissue samples. Moreover, the reliability of the prediction results is improved by the way of multiple database prediction, especially the support of laboratory data. However, it has limitations. First and foremost, all of the datasets for exploration were obtained from the GEO online database. Further experiments should be performed to verify our results and explore their potential clinical applications. Secondly, due to the relatively low incidence of the disease in the population and the scarcity of sequencing data, we were unable to conduct large-scale exploration and verification to make our conclusion more convincible. Anyway, our findings focused on the functions of ncRNAs in SSc and filtered convincible ceRNA interaction pairs. It shed new light on the development of SSc, although the exact molecular mechanism of candidate genes and functional pathways in SSc still needs to be further explored.

## Data Availability Statement

The original contributions presented in the study are included in the article/supplementary material, further inquiries can be directed to the corresponding author/s.

## Author Contributions

Y-MY and QW designed the study. Y-MY did the statistical analyses and prepared figures. Y-MY, J-NZ, L-WW, Q-WR, Q-RY, DG, and QW reviewed the results and wrote the manuscript. All authors have made an intellectual contribution to the manuscript, and read and approved the final manuscript.

## Conflict of Interest

The authors declare that the research was conducted in the absence of any commercial or financial relationships that could be construed as a potential conflict of interest.

## Footnotes

1 http://www.ncbi.nlm.nih.gov/geo/
